# The relationship between reported fever and *Plasmodium falciparum *infection in African children

**DOI:** 10.1186/1475-2875-9-99

**Published:** 2010-04-19

**Authors:** Emelda A Okiro, Robert W Snow

**Affiliations:** 1 Malaria Public Health & Epidemiology Group, Centre for Geographic Medicine Research - Coast, Kenya Medical Research Institute/Wellcome Trust Research Programme, PO Box 43640, 00100 GPO, Nairobi, Kenya; 2 Centre for Tropical Medicine, Nuffield Department of Clinical Medicine, University of Oxford, CCVTM, Oxford OX3 7LJ, UK

## Abstract

**Background:**

Fever has traditionally served as the entry point for presumptive treatment of malaria in African children. However, recent changes in the epidemiology of malaria across many places in Africa would suggest that the predictive accuracy of a fever history as a marker of disease has changed prompting calls for the change to diagnosis-based treatment strategies.

**Methods:**

Using data from six national malaria indicator surveys undertaken between 2007 and 2009, the relationship between childhood (6-59 months) reported fever on the day of survey and the likelihood of coincidental *Plasmodium falciparum *infection recorded using a rapid diagnostic test was evaluated across a range of endemicities characteristic of Africa today.

**Results:**

Of 16,903 children surveyed, 3% were febrile and infected, 9% were febrile without infection, 12% were infected but were not febrile and 76% were uninfected and not febrile. Children with fever on the day of the survey had a 1.98 times greater chance of being infected with *P. falciparum *compared to children without a history of fever on the day of the survey after adjusting for age and location (OR 1.98; 95% CI 1.74-2.34). There was a strong linear relationship between the percentage of febrile children with infection and infection prevalence (R^2 ^= 0.9147). The prevalence of infection in reported fevers was consistently greater than would be expected solely by chance and this increased with increasing transmission intensity. The data suggest that in areas where community-based infection prevalence in childhood is above 34-37%, 50% or more of fevers are likely to be associated with infection.

**Conclusion:**

The potential benefits of diagnosis will depend on the prevalence of infection among children who report fever. The study has demonstrated a predictable relationship between parasite prevalence in the community and risks of infection among febrile children suggesting that current maps of parasite prevalence could be used to guide diagnostic strategies in Africa.

## Background

The aetiology of fevers in malaria endemic areas has been the subject of considerable basic and applied public health research for many years. *Plasmodium falciparum *is not the only cause of fever and only one of many pathogens that cause identical pyrogenic responses in young African children [1-6]. Although fever usually has a high sensitivity for the diagnosis of malaria it suffers from poor specificity and critically depends on the prevalence of both asymptomatic infection and the overall prevalence of fever. For decades fever has served as the entry point for presumptive treatment of malaria in African children who, if left untreated, run the risks of severe complications and death. Presumptive treatment of all fevers has, therefore, been the most risk-adverse approach to managing "malaria" across Africa and is enshrined in the recommendations proposed by the Integrated Management of Childhood Illnesses (IMCI) [7-12]. There is, however, increasing evidence that the intensity of *P. falciparum *transmission is declining across many parts of Africa [13] and this transition now supports lower infection prevalence in childhood that may alter the sensitivity and specificity of a fever history as a marker of disease [9,14]. The WHO have now moved away from presumptive treatment in Africa to one that recommends parasitological diagnosis [15]

There is an increasing body of data derived from national household sample surveys where the presence of reported fever among children below the age of five and malaria infection are recorded using interviews with caretakers and rapid diagnostic tests (RDTs). An evaluation of these data provides a new opportunity to assess the proportion of childhood fevers likely to be associated with malaria infection across a range of contemporary malaria transmission settings in Africa.

## Methods

### Data description

Malaria indicator surveys (MIS) are promoted as tools to monitor national level progress toward milestones set by national malaria control programmes and the Roll Back Malaria movement on the coverage of key malaria prevention and disease management strategies [16]. These surveys are either stand-alone malaria surveys or included as modules within broader Demographic and Health Surveys (DHS) or UNICEF's Multiple Indicator Cluster Surveys (MICS). Surveys are designed to be nationally representative and powered to provide adequate precision in coverage of interventions, such as insecticide-treated nets, at the first-level administrative unit (Province or Region) and typically cover sample sizes of approximately 3,000 - 6,000 households derived from a two-stage cluster sample design. Data from six national malaria indicator surveys undertaken between 2007 and 2009 in Djibouti, Kenya, Namibia, Angola, Liberia and Senegal were used and cover a broad range of malaria transmission conditions typical of the Horn, West, East and Southern Africa (Table 1). All surveys included questions on the presence of fever on the day of the survey among children aged between six and 59 months of age through interviews with mothers or guardians. This was an important inclusion criterion in the selection of surveys as it allows for the simultaneous comparison of reported illness and the presence or absence of infection. All surveys employed RDTs to identify infection during the survey (RDT types shown in Table 1). In some cases these were checked against microscopy, but this was not a uniform practice across all surveys and, therefore, only the results of RDT positivity have been used.

**Table 1:** Summary of national households surveys with data on Fever today: data assembled to define relationship between prevalence of fever and infection among children 0.5-4 years from national sample surveys in 6 African countries

Country Source	ADMIN UNITS	Survey Date	RDT used	Total Seen
Angola [41]	19^1^	Nov 2006-Apr 2007	Paracheck Pf	1433
Djibouti [42]	6^2^	Dec 2008-Jan 2009	Hexacon & ParaHit	847
Kenya [43]	7	Jun 2007-Jul 2007	Paracheck Pf	4755
Liberia [44]	15^3^	Dec 2008-Mar 2009	Paracheck Pf	1547
Namibia^5^	9^4^	May 2009-Jun 2009	Paracheck Pf	1074
Senegal [45]	11	Nov 2008-Feb 2009	Paracheck Pf	3242

Notes:

^1^Data from Angola is reportedin three recognized malaria epidemiologic regions: Hyperendemic which covers six provinces: Cabinda, Uige, Kwanza N., Malange, Lunda N., Lunda S.; Mesoendemic Stable which includes: Zaire, Luanda, Bengo, Benguela, Kwanza S., Huambo, Bié and Mesoendemic Unstable which includes: Moxico, Kuando, Kubango, Kunene, Huila, Namibe

^2^Data from Djibouti has been split into two regions the Capital city which includes Arta and Djibouti Ville and Rural which includes the regions of Ali Sabieh, Ddikhil, Obock and Tadjourah.

^3^Data from Liberia is reported in six regions: Greater Monrovia; and five regional groupings formed by grouping the 15 counties: North Western: (Bomi, Grand Cape Mount, Gbarpolu); South Central: Montserrado (outside Monrovia), Margibi, Grand Bassa; South Eastern A: River Cess, Sinoe, Grand Gedeh; South Eastern B: River Gee, Grand Kru, Maryland; and North Central: Bong, Nimba, Lofa

^4 ^For Namibia we combined data into 4 regional groups: group 1: Caprivi and neighbouring Kavango; group 2: Otjozondjupa and Omaheke; group 3: Oshikoto, Oshana, Ohangwena and Omusati and group 4 with data from Kunene

^5 ^Petruni; personal communication.

To provide an analysis across malaria risk classifications we have reconstructed individual level data to represent the administrative units used principally as part of the multi-stage sampling, hereafter referred to as ADMIN1 level. These ADMIN1 units represented provinces or regions in Kenya and Senegal. In Angola, sampling was designed to represent three recognized malaria epidemiologic regions: hyper-endemic, meso-endemic stable and meso-endemic unstable and these divisions were used within the present analysis. In Liberia, survey data were reported for Monrovia and each of five regional groupings that do not correspond directly to national administrative boundaries. Due to very low parasite prevalence in Djibouti, data have been summarized at larger spatial resolutions as the Capital and Rural Djibouti. For similar reasons, the data to four regional groupings in Namibia were aggregated as shown in Table 1.

### Analysis

Data were assembled across each of the 33 ADMIN1 units assembled from the six national sample surveys. Each of the 33 sites was classified according to the overall prevalence of *P. falciaprum *infection (*Pf*PR) among all surveyed children and those that were afebrile. First, individual child level data were used to investigate the risks of infection if the child was reported as having a fever using the Mantel-Haenszel estimate of the odds ratio that adjusted for the age of the child and the overall prevalence of infection within each of the ADMIN1 units. Second, the relationships between the percentage of febrile children with infection was examined against the overall prevalence of infection among all children sampled in each ADMIN1 unit. In addition the prevalence of infection among those who did not report a fever on the day of the survey was examined against the percentage of febrile children with infection thus specifically excluding the children in the dependent variable from the predictor (x-axis). In both cases it is assumed that true zero infection prevalence would equate to zero prevalence in febrile children to anchor the association through the X-Y intercept (i.e. using a zero intercept). Confidence intervals (CIs) around the best-fit association between infection prevalence and prevalence of infection in febrile children were computed providing the boundaries of all possible linear fits to the data. Linear prediction plots were defined and included CIs around the predicted line using the *twoway lfitci *command in Stata. All data were analysed using STATA version 11.0 (StatCorp, College Station, Texas)

## Results

Data were assembled on fever and infection prevalence among 16,903 children surveyed at 33 different locations across six countries. The surveys recorded a total of 2,071 (range 10-192 per site) children with a fever on the day of survey and 2,471 (range 0-313 per site) children with a positive RDT result for *P. falciparum*. 535 (3%) of the children were both febrile and infected, 1,536 (9%) were febrile without evidence of infection, 1,936 (12%) were infected but were not reported as febrile, and 12,896 (76%) were neither infected nor febrile. The 33 sampling units surveyed represented a wide range of transmission intensities ranging from *P. falciparum *parasite prevalence among all sampled children 0.5 to 4 years (*Pf*PR_0.5-4_) values of 0% in rural Djibouti to as high as 44.4% in North Central, Liberia. 13 sites were identified as having a *Pf*PR_0.5-4 _<5%, 18 sites were characterised as having a *Pf*PR_0.5-4 _within 5-39% and only two sites were located in the highest transmission class with *Pf*PR_0.5-4 _≥ 40%. The median parasite prevalence across the entire range was 11.74% (IQR: 2.78-22.94%). The median fever prevalence across the entire data series range was 9.25% (IQR: 6.50-16.43%).

**Figure 1:**
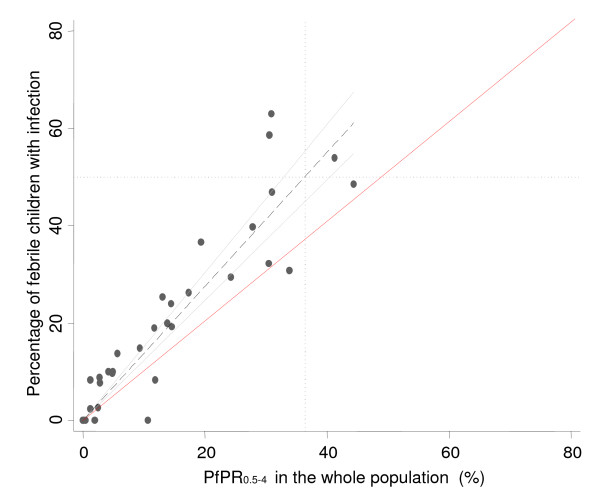
**Graph showing the least-square linear regression line *y *= 1.376*x *(black dashed lines) over a scatter plot showing the continuous relationship between the percentage of febrile children with infection and infection prevalence among all children and plotted with the 95% confidence interval**. Two lines surrounding the best-fit line (grey solid lines) define the confidence interval. We also show the point at which febrile infections exceeds 50% and the corresponding value for infection prevalence (black dotted horizontal and vertical lines) including a line illustrating the 50%-50% random chance of symptomatic infection.

**Figure 2:**
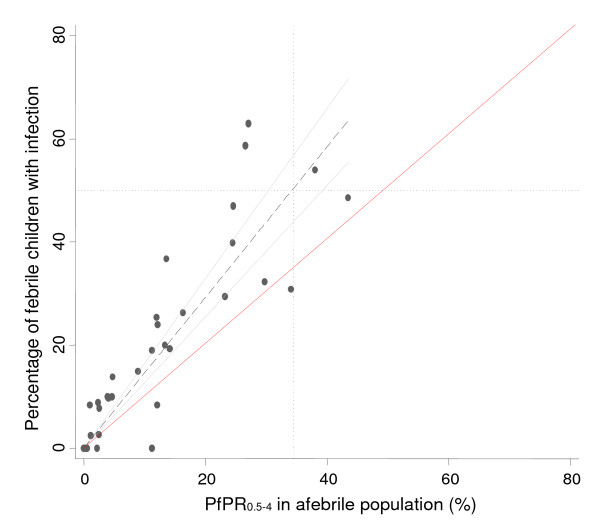
**Graph showing the least-square linear regression line *y *= 1.460*x *(black dashed lines) over a scatter plot showing the continuous relationship between the percentage of febrile children with infection and infection prevalence among children who did not report a fever and plotted with the 95% confidence interval**. Two lines surrounding the best-fit line (grey solid lines) define the confidence interval. We also show the point at which febrile infections exceeds 50% and the corresponding value for infection prevalence among the asymptomatic population (black dotted horizontal and vertical lines) including a line illustrating the 50%-50% random chance of symptomatic infection.

Children with a reported fever on the day of the survey had a 1.98 times greater chance of being infected with *P. falciparum *compared to children without a history of fever on the day of the survey after controlling for age and ADMIN1 residence (95% CI 1.74-2.34; P < 0.0001). The continuous relationship between the percentage of febrile children with infection and infection prevalence (parasite prevalence) among all children is shown in Figure 1. The best-fit association was linear when anchored at the X-Y intercept (y = 1.376×) and the positive linear relationship was highly significant (R^2 ^= 0.9147, p < 0.0001). Comparing this correlation to the 50%-50% equivalent chance of fevers and all children being infected (red line in the Figure 1) shows that the prevalence of infection in reported fevers was consistently greater than would be expected purely by chance and increased with increasing *Pf*PR_0.5-4_. Figure 1 highlights that in areas where *Pf*PR_0.5-4 _is greater than 37% more than 50% of fevers can be predicted to be infected. This comparison was repeated using only the prevalence of infection among afebrile children as a marker of transmission intensity (Figure 2) providing similar results to those of Figure 1 (R^2 ^= 0.8741, p < 0.0001) and a prevalence of infection among asymptomatics of approximately 34% corresponding to 50% of fevers infected with *P. falciparum*.

## Discussion

Recognizing the combined needs to mitigate the threats of drug pressure on the emergence of drug resistance, the changing malaria epidemiology in Africa and the need to improve the way febrile children are managed clinically has provided support for calls to move away from presumptive treatment of African children [14]. Children managed as malaria without parasites have a high probability of poor clinical outcomes because other causes of illness are missed [17-19]. To consider this in more depth this paper examines the relationships between reported fever symptoms and the risks of being infected using data from six national household sample surveys. Among 16,903 children, 12% were reported as having a fever on the day of the survey of which approximately 26% had evidence of *P. falciparum *infection as judged by an RDT and 74% were aparasitaemic. Clearly, among these communities the use of fever alone as a presumptive prompt for anti-malarial treatment would result in a huge over-treatment burden. Conversely, among the 15% of children with evidence of infection they were almost twice as likely to be febrile on the day of investigation when compared to children without infection suggesting that infection in these areas is a reasonable predictor of symptoms. These results are specific to the range of transmission conditions represented by the six national surveys and did not include many sites at the highest transmission intensity. Nevertheless there is increasing evidence that low parasite prevalence is a common feature of most of Southern Eastern and Horn of Africa countries [13] and those previously moderately high transmission areas are transitioning to low parasite prevalence coincidental with scaled prevention [20-26]. In these areas, it is concluded that infection with *P. falciparum *is very likely to result in symptoms that include fever, presumably because of poorly developed functional immunity through the first five years of life, but fever alone remains a poor discriminator of malaria infection suggesting that all fevers should be tested to confirm or refute the role of malaria in the febrile presentation.

These findings are not new. However, previous studies have largely been undertaken among clinic attending populations at a time when asymptomatic parasite prevalence was higher across more countries in Africa. The results presented here are of randomly selected children resident within a wide range of communities experiencing very different malaria transmission conditions. Figures 1 and 2 suggest that there is a point on the transmission intensity X-axis, approximately 34-37%, where 50% or more of fevers are likely to be associated with infection, a point at which RDT use may become less cost-efficient. This cut-off has important implications for the use of currently available *P. falciparum *transmission maps for Africa which are able to provide a prediction with definable uncertainty of malaria prevalence in the community [13] and could be used to guide where parasitological diagnosis of childhood fevers is most cost-efficient. From the predictions made for 2007, over 48.38 million children aged less than five years live in areas where parasite prevalence is less than 40% (which is close to the figure of 37% shown in Figure 1), representing 44.5% of children in *P. falciparum *malaria endemic countries in Africa.

The analysis comes with several caveats. First, the application of community-survey data on reported fever prevalence assumes that fever reporting on the day of the survey is accurate and similar to what might be reported by mothers of sick children attending clinic. The recognition of fever as a morbid event to interviewers or health workers varies within and between communities who have developed an elaborate cultural vernacular to describe fever [27-33]. Not all fevers reported during household sample surveys seek treatment [34] and those fevers that do attend clinic may have different characteristics to those reported in the community. Importantly, health workers may probe for more precise descriptions of fever than field staff recording symptom histories during household surveys. Second, the results apply to fever prevalence in the community and not at clinic. It is recognized that the proportion of fevers presenting to clinic harboring infection may be higher than among fevers reported in the community and requires further investigation. Finally, only the presence/absence of infection by RDT has been examined rather than attributable fractions necessary for clinical definitions within clinical trials based upon parasite density criteria [8,35-39]. Consequently it is perhaps not surprising that the higher the background infection prevalence the more likely a fever is associated with infection. Figures 1 and 2 are, therefore, not biological relationships of clinical risk, but pragmatic associations of fever and RDT positivity that are linear and predictable with direct implications for the rolling out of RDT in clinic settings across a range of transmission conditions. However, these results will also depend upon the range of sensitivities and specificities of currently available RDTs proposed for clinic use[40].

## Conclusion

Africa has made significant progress in the control of malaria and this has led to a call for diagnosis-based treatment strategies. The cost-effectiveness of imposing diagnosis for febrile children will depend on the prevalence of infection among those who report fever. The data presented here indicate a predictable relationship between parasite prevalence in the community and risks of infection among febrile children implying that mapped estimates of parasite prevalence can be used to guide diagnostic strategies in Africa.

## Conflict of interests

The authors declare that they have no competing interests.

## Authors' contributions

EAO assembled all the data, restructured and analysed the data and wrote the manuscript. RWS was responsible for the project and its overall scientific management, interpretation and preparation of the final manuscript. Both authors read and approved the final manuscript.
